# Diazonium-Modified Screen-Printed Electrodes for Immunosensing Growth Hormone in Blood Samples

**DOI:** 10.3390/bios9030088

**Published:** 2019-07-17

**Authors:** Nan Li, Ari M. Chow, Hashwin V. S. Ganesh, Melanie Ratnam, Ian R. Brown, Kagan Kerman

**Affiliations:** 1 Department of Physical and Environmental Sciences, University of Toronto Scarborough, Toronto, ON M1C 1A4, Canada; 2 Centre for the Neurobiology of Stress, Department of Biological Sciences, University of Toronto Scarborough, Toronto, ON M1C 1A4, Canada

**Keywords:** immunosensor, electrochemical impedance spectroscopy, growth hormone, real samples, diazonium grafting

## Abstract

Altered growth hormone (GH) levels represent a major global health challenge that would benefit from advances in screening methods that are rapid and low cost. Here, we present a miniaturized immunosensor using disposable screen-printed carbon electrodes (SPCEs) for the detection of GH with high sensitivity. The diazonium-based linker layer was electrochemically deposited onto SPCE surfaces, and subsequently activated using covalent agents to immobilize monoclonal anti-GH antibodies as the sensing layer. The surface modifications were monitored using contact angle measurements and X-ray photoelectron spectroscopy (XPS). The dissociation constant, K_d_, of the anti-GH antibodies was also determined as 1.44 (±0.15) using surface plasmon resonance (SPR). The immunosensor was able to detect GH in the picomolar range using a 20 µL sample volume in connection with electrochemical impedance spectroscopy (EIS). The selectivity of the SPCE-based immunosensors was also challenged with whole blood and serum samples collected at various development stages of rats, demonstrating the potential applicability for detection in biological samples. Our results demonstrated that SPCEs provided the development of low-cost and single-use electrochemical immunosensors in comparison with glassy carbon electrode (GCE)-based ones.

## 1. Introduction

In an immunosensor, the integration of the recognition element with the signal transducer is usually achieved by a chemical layer modification that enables the immobilization of antibodies. Aryl diazonium salts have become increasingly popular given their ease of use for modifying a wide variety of surfaces and their stability as a chemical linker [1,2,3,4]. Eissa et al. [5] reported the electrochemical modification of graphene-modified screen-printed carbon electrodes (SPCEs) with 4-nitrophenyl diazonium salt, which enabled the covalent attachment of antibodies for the detection of a milk allergen, β-lactoglobulin. Eissa and Zourob [6] have also reported the development of an electrochemical competitive immunosensor for the detection of okadaic acid in shellfish. Graphene modified SPCEs were functionalized by the electrochemical reduction of in situ generated 4-carboxyphenyl diazonium salt in acidic aqueous solution [6]. The sensitive detection of egg allergen ovalbumin was also achieved with a detection limit of 0.83 pg/mL in phosphate buffer solution (PBS) using graphene modified SPCEs with a carboxyphenyl film on the graphene surface [7]. SPCEs can be mass-produced at low-cost, and each experiment can be performed on a fresh and analogous surface to prevent possible cross-contamination issues [8]. Each SPCE can be disposed after use and requires small volumes of reagents for measurement. Furthermore, current advances in instrumentation have allowed SPCEs to become compatible with cellphone-size portable devices for convenient on-site measurements [9].

GH is a peptide hormone with two isoforms (22 kDa and 20 kDa) released from the somatotroph cells in the anterior pituitary gland, serving multiple functions in many tissue targets [10]. GH plays a critical role in the regulation of blood glucose, longitudinal bone growth, and enhancement of muscle mass [11]. Deficiencies in GH can have severe developmental consequences, such as hypoglycemia in newborns, stunted growth in childhood, and physical/psychological symptoms well into adulthood [12,13]. On the other hand, over expression of GH can result in gigantism, acromegaly, and impaired glucose tolerance [14,15,16]. Beyond a clinical setting, GH doping is observed extensively in professional athletes to enhance overall tissue maintenance, repair, and muscle growth [17]. The use of synthetic GH as an ergogenic drug has become increasingly prevalent, which resulted in its ban from professional athletes by the World Anti-Doping Agency (WADA) [18]. To face these major global health challenges, advances in GH screening are required to facilitate early detection and treatment [19]. At present, reported GH screening methods include enzyme-linked immunosorbent assays (ELISA) [20], radioimmunoassays (RIA) [21], mass spectrometry [22], and surface plasmon resonance (SPR) [23]. Current issues concerning these methods include sensitivity, specificity, cost and time. There is an urgent need for an ultrasensitive screening procedure for the detection of GH that is rapid and low cost [24,25,26,27,28,29,30]. Our immunosensor provides an economical approach to a miniaturized electrochemical system utilizing SPCEs. As a model system, we modified SPCEs with anti-GH antibodies using diazonium grafting and compared the analytical characteristics of SPCE-based immunosensors with similarly prepared GCE (glassy carbon electrode)-based ones. Then, we demonstrated the detection of GH in blood and serum samples using SPCE-based immunosensors in comparison with a commercially available kit.

## 2. Materials and Methods

### 2.1. Chemicals and Reagents

Rat growth hormone (GH), full-length protein (ab68388) and monoclonal mouse anti-growth hormone (GH) antibody (ab9821) were obtained from Sigma-Aldrich (Oakville, ON, Canada). Potassium dihydrogen orthophosphate (KH_2_PO_4_), dipotassium orthophosphate (K_2_HPO_4_), 1-ethyl-3-(3-dimethylaminopropyl) carbodiimide hydrochloride (EDC), N-hydroxysuccinamide (NHS), potassium ferricyanide (K_4_Fe(CN)_6_), potassium ferrocyanide (K_3_Fe(CN)_6_), 4-methoxybenzenediazonium tetrafluroborate, acetonitrile (HPLC grade), and bovine serum albumin (BSA) were obtained from Sigma-Aldrich (Oakville, ON, Canada). Tetraethylammonium tetrafluoroborate was purchased from Alfa Aesar (Mississauga, ON, Canada). All solutions were prepared using ultra-pure water from a Cascada LS water purification system (Pall Co., Port Washington, NY, USA) at 18.2 MΩ. Male Sprague Dawley rats (Charles River, MA, USA) were anesthetized with isoflurane and then maintained under isofluorane by a vaporizer with a nose cone attachment. Blood was drawn by cardiac puncture in the right ventricle. The blood samples were subsequently analyzed using ELISA and our electrochemical immunosensor for growth hormone levels. For control studies, the GH sample concentration used for the blood and plasma was 100 pg/mL. An undiluted sample (also coined as blood stock or plasma stock) indicates that the blood or plasma sample was used as received without any further dilutions using buffer solutions. All procedures using animals were approved by the Animal Care Committee of the University of Toronto and were in accordance with the guidelines and codes established by the Canadian Council on Animal Care.

### 2.2. Electrode Preparation and Modification

**Figure 1:**
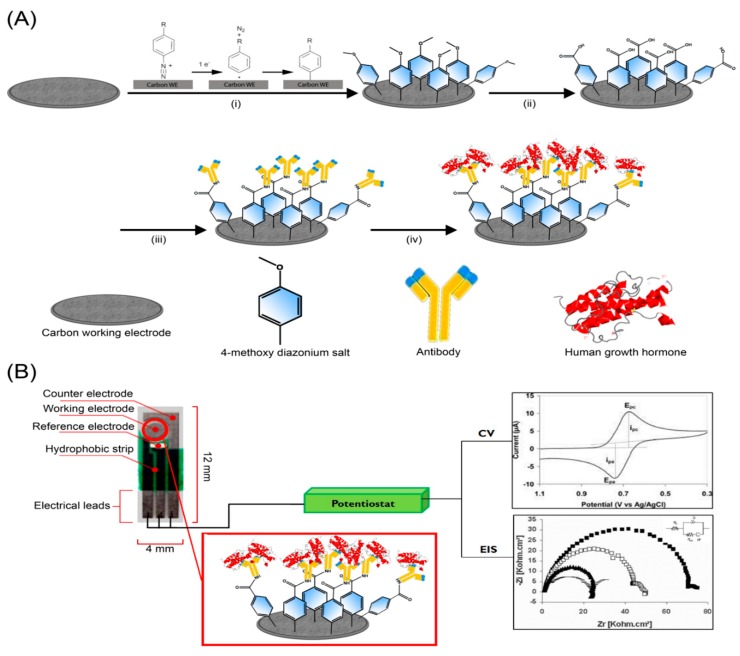
Conceptual illustration of the impedimetric detection of rat growth hormone (GH). (**A**) A film of 4-methoxybenzenediazonium tetrafluroborate (4-MBD) was immobilized on an electrode surface by electrodeposition (i); surface-confined 4-MBD molecules were electrochemically oxidized to carboxylic acid groups (ii) that were activated with 1-ethyl-3-(3-dimethyl-aminopropyl) carbodiimide (EDC) and N-hydroxysuccinimide (NHS) to allow subsequent covalent immobilization of antibodies via lysine residues (iii); GH is captured with antibodies on the surface (iv). (**B**) screen-printed carbon electrodes (SPCE) of dimension 4 × 12 mm in length with carbon ink-based working and counter electrodes in connection with a silver ink-based reference electrode. Electrochemical impedance spectroscopy (EIS) and cyclic voltammetry (CV) were employed to characterize SPCE and glassy carbon electrode (GCE) surfaces.

A glassy carbon electrode (GCE, CH Instruments, Austin, TX, USA) was polished for 2 min using 1.0, 0.3, and 0.05 μm alumina. The electrode was rinsed with ultrapure water and sonicated for 5 min between each polishing step to remove any alumina present on the surface. Screen-printed carbon electrodes (SPCEs, DEP-Chip EP-N) were purchased from BioDevice Technology Ltd. (Ishikawa, Japan). As shown in Figure 1B, SPCEs had a carbon ink-based working electrode surface that enabled working with small volumes of reagents and samples (1–20 µL). The counter and reference electrodes were printed using carbon and silver ink, respectively. The electrical contacts were protected from sample solutions with a hydrophobic coating as shown in the inset of Figure 1B.

The surface modification of GCEs and SPCEs was performed following the same procedures of electrodeposition. Briefly, aryl diazonium salt (1 mM) in acetonitrile was prepared and de-aerated for 15 min. Electrodeposition was performed using tetraethylammonium tetrafluoroborate as the electrolyte with CV. A scan rate of 100 mV/s was applied for two cycles between +1.0 V and −1.0 V, and then rinsed with copious amounts of electrolyte and water [1,2]. Next, the electrode was exposed to a biasing potential of +1.0 V for 1 min in 50 mM KH_2_PO_4_/K_2_HPO_4_ (PBS) at pH 7.4. The activation of exposed carboxylic acid groups on the surface was accomplished using 10 mM EDC and 40 mM NHS for 1 h and rinsed with PBS [4]. The antibodies were incubated on the electrode surface, to allow covalent attachment between the free amine groups of lysine residues in the Fc region and the carboxyl groups on the diazonium grafted surfaces for 18 h at 4 °C, then rinsed with PBS. GH sample solutions were prepared at various concentrations in PBS and used as the target analyte. An aliquot (10 µL) of each sample solution (real samples or PBS spiked with the desired amount of GH) was added to the electrode surface and incubated for 30 min at room temperature with moderate shaking, to ensure contact of the GH with the surface-confined antibodies.

### 2.3. Electrochemistry

**Figure 2:**
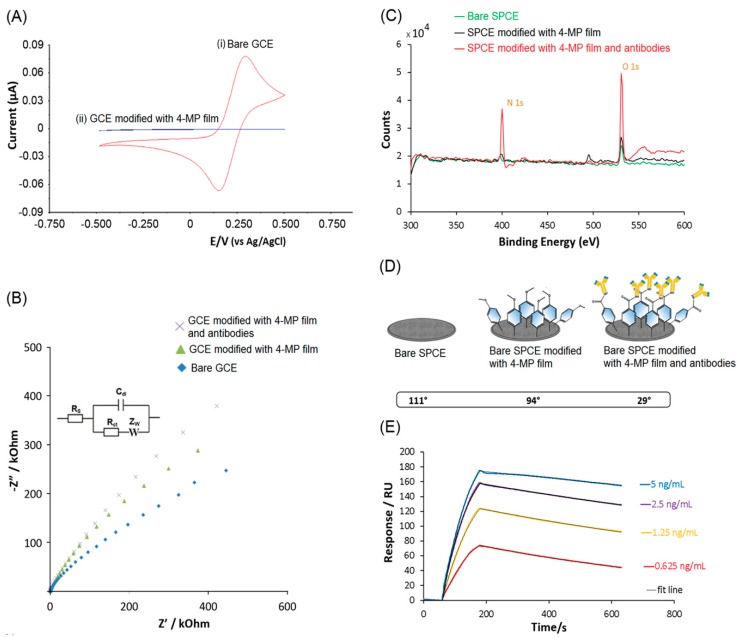
Surface characterization of electrode surface modifications. (**A**) Cyclic voltammogram of (i) bare glassy carbon electrode (GCE) and (ii) 4-methoxyphenyl (4-MP) film modified GCE using 10 mM [Fe(CN)_6_]^3−/4−^ in PBS with 100 mM KCl at 100 mV/s as described in the Materials and Methods section. (**B**) EIS measurements demonstrated with Nyquist plot (−Z″ vs Z′) and fitted with Randles equivalent circuit for the characterization of bare GCE, 4-MP film-modified electrodes and antibody-modified electrodes. (**C**) XPS-based characterization of bare screen-printed carbon electrode (SPCE), 4-MP film modified electrode, and antibody modified electrode. (**D**) Contact angle measurements of bare SPCE, 4-MP film modified electrode, and antibody-modified electrode. (**E**) SPR-based immunosensor measurements to determine the K_d_ of antibody with targeted GH as the analyte. SPR responses increased as the concentration of GH increased from 0.625 ng/mL (red line), 1.25 ng/mL (yellow line), 2.5 ng/mL (purple line) to 5 ng/mL (blue line). SPR sensorgrams were modeled with a fit line (black line) to determine the binding parameters. The sensorgram displays the average data of three consecutive measurements for each concentration of GH on renewed sensorchip surfaces (n = 3).

Cyclic voltammetry (CV) and electrochemical impedance spectroscopy (EIS) were performed using a μAutolabIII Electrochemical Analyzer (Metrohm, Utrecht, The Netherlands) in conjunction with its general-purpose electrochemistry software and a frequency response analyzer (Metrohm, Utrecht, The Netherlands). CV and EIS were measured with 10 mM [Fe(CN)_6_]^3−/4−^ in PBS with 100 mM KCl at room temperature using a three-electrode system with GCE as the working electrode, a Pt wire as the counter electrode, an Ag/AgCl reference electrode and also SPCE as depicted in the inset of Figure 2. CV measurements were performed before and after aryl diazonium salt modification at a scan rate of 100 mV/s between −0.5 V and +0.5 V. EIS was performed with a frequency range from 100 kHz to 100 mHz at a biasing voltage of 0.20 V.

### 2.4. Surface Plasmon Resonance (SPR)

All SPR experiments were performed using a Biacore X100 system (GE Healthcare, Mississauga, ON, Canada) with a CM5 sensorchip. Experiments were conducted at 25 °C and the SPR running buffer (SPR running buffer, 0.01 M HEPES, 0.15 M NaCl, 0.05 mM EDTA, 0.05% surfactant P20, pH 7.4) was sterile filtered (0.2 μm). Two flow cells of the sensorchip were used, one (reference flow cell, FC-1) to detect the non-specific adsorption for background subtraction, and the other one (detection flow cell, FC-2) was used to detect the specific binding of GH. CM5 sensorchips contain carboxymethylated dextran covalently attached to Au surfaces. The anti-GH antibodies were covalently coupled onto the CM5 sensorchip surfaces using the Amine Coupling Kit (GE Healthcare, Mississauga, ON, Canada), following the standard covalent attachment protocol using EDC and NHS in a similar fashion as described in the preparation of electrochemical biosensors in Section 2.2. The target GH samples were injected into both flow cells using four different concentrations at 30 μL/min for 2 min. The setup was fully automated using the Biacore X100 software (GE Healthcare, Mississauga, ON, Canada). All concentrations of the target GH were performed in triplicates, with zero concentration blanks before and after each injection of the sample. The binding affinity of the interaction was determined using Biacore Evaluation Software (GE Healthcare, Mississauga, ON, Canada).

### 2.5. X-Ray Photoelectron Spectroscopy (XPS)

XPS spectra were recorded using a Physical Electronics (PHI) Quantera II spectrometer (Laval, QC, Canada) equipped with an Al anode source for X-ray generation and a quartz crystal monochromator for focusing the generated X-rays. A monochromatic Al K-α X-ray (1486.7 eV) source was operated at 50 W and 15 kV, and a pass energy of 280 eV was used to obtain all collected survey data. All spectra were obtained at 45° take off angles, and a dual beam charge compensation system was used to neutralize all samples. The system base pressure was no higher than 1.0 × 10^−9^ Torr, with an operating pressure that did not exceed 2.0 × 10^−8^ Torr. The instrument was calibrated using a sputter-cleaned piece of Ag, where the Ag 3d5/2 peak had a binding energy of 368.3 ± 0.1 eV and full width at half maximum for the Ag 3d5/2 peak was at least 0.52 eV. Data manipulation was performed using PHI MultiPak Version 9.5.1.0 software (Laval, QC, Canada).

### 2.6. Contact Angle Goniometry

Contact angles of modified surfaces were measured using a Future Digital Scientific OCA35 system (Westbury, NY, USA). Milli-Q water (18.2 Ohms) was used to determine the contact angles. The static sessile drop method was used and a small droplet (~0.2 μL) of Milli-Q water was placed on the sample surface. A picture was captured for each droplet on modified surfaces, and the contact angle was calculated using Young’s equation.

### 2.7. ELISA-Based Detection

GH detection kit was purchased from EMD Millipore (Etobicoke, ON, Canada). In the sandwich-based assay, the GH samples were captured by the pre-tittered anti-GH polyclonal antibodies on the 96-well plate, and then, the binding of a second biotinylated anti-GH polyclonal antibody would form a “sandwich” by capturing the target protein on the surface. Streptavidin-conjugated horseradish peroxidase (HRP) was then exposed to the biotinylated antibodies. Upon addition of TMB (3,3′,5,5′-tetramethylbenzidine) substrate, the quantitative detection was achieved under acidic conditions with the formation of yellow-colored product at 450 nm. The concentration of GH in blood and plasma samples was derived by interpolation from a calibration curve generated in the assay, with reference standards of known concentrations of rat GH at 0.07, 0.21, 0.62, 1.9, 5.6, 16.7, and 50 ng/mL. We were able to determine the GH concentration in blood samples using the calibration plot that was constructed using the standard additions of known concentrations of GH in blood samples. We have also used this ELISA-based commercial kit to confirm our EIS data obtained with the standard addition method.

## 3. Results and Discussion

Following the chemical reactions as illustrated in Figure 1A, 4-methoxyphenyl (4-MP) film was formed through electrodeposition (i). Upon electrochemical grafting, liberation of nitrogen gas occurred, forming an aryl radical intermediate. The radical then formed a covalent bond with the carbon surface with high stability over time [31,32,33]. A conditioning potential of +1.0 V (vs. Ag/AgCl) was applied to form carboxylic acid on the film (ii). Subsequently, the carboxylic acid groups were activated with 1-ethyl-3-(3-dimethylaminopropyl) carbodiimide (EDC) and N-hydroxysuccinamide (NHS) (iii) to immobilize the antibodies by forming amide bonds with the lysine residues of the Fc region. Various concentrations of GH could be detected using the antibodies immobilized on the surface (iv) through antibody–antigen interactions. As shown in Figure 1B, both cyclic voltammetry (CV) and electrochemical impedance spectroscopy (EIS) [34,35,36,37] were used to characterize the modifications on carbon surfaces.

As shown in Figure 2A-i, the bare GCE displayed prominent oxidation and reduction peaks for the [Fe(CN)_6_]^3-/4-^ redox couple at 0.28 V and 0.16 V, respectively. The electrodeposition of 4-MP film resulted in a significant decrease in cyclic voltammograms, which suggested that the 4-MP film created an insulating layer that suppressed the reaction of the [Fe(CN)_6_]^3/4-^ redox couple on the surface (Figure 2A-ii).

EIS was used to detect the interfacial properties for bare GCE, 4-MP film-modified GCE, and antibody-modified GCE. As shown in Figure 2B, the impedimetric measurements were demonstrated with Nyquist plots as the sum of the Z′ and Z″. The Z′ (real Z) accounts for double-layer resistance and Z″ (imaginary Z) accounts for capacitance. The Randles equivalent circuit shown in the inset of Figure 2B was selected to reflect the electrochemical process. The equivalent circuit includes the ohmic resistance of the electrolyte solution, R_s_, and the Warburg impedance, Z_w_, resulting from the diffusion of ions from the bulk electrolyte to the interface. The electrode double-layer capacitance, C_dl_, and the charge transfer resistance, R_ct_, depends on the dielectric and insulating properties at the interface. R_ct_ values continued to increase with the step-wise modifications with 4-MP film and then antibodies on the electrode surfaces.

The increase in R_ct_ was consistent with the previous CV data suggesting the aryl diazonium salt film had an insulating property. A further increase in R_ct_ suggested that antibodies were immobilized onto the GCE surface. The EIS results were confirmed using XP. As shown in Figure 2C, the increases in O 1s’ counts—shown in green lines and red lines—were contributed to by the oxygen atoms in the 4-MP film and antibodies, and the significant increase in N 1s’ counts was attributed to the primary amine groups of the antibodies, suggesting the immobilization of the antibodies on the SPCE surface. As shown in Figure 2D, the contact angle measurement had a small decrease after the immobilization of 4-MP film, and then, a significant decrease was observed after the immobilization of antibodies. The contact angle measurements were in agreement with the XPS results (Figure 2C), where the small increase in O 1s after immobilization of 4-MP film, followed by a significant increase in O 1s after the immobilization of antibodies, would contribute to the decreasing hydrophobicity of the electrode surface. In order to determine the binding dissociation constant, K_d_, value of the anti-GH antibody, SPR (Figure 2E) was performed using anti-GH-modified CM5 sensorchips. The anti-GH antibodies that were covalently immobilized on CM5 sensorchips displayed a strong binding affinity towards GH at 1.44 (±0.15) nM (n = 3).

**Figure 3:**
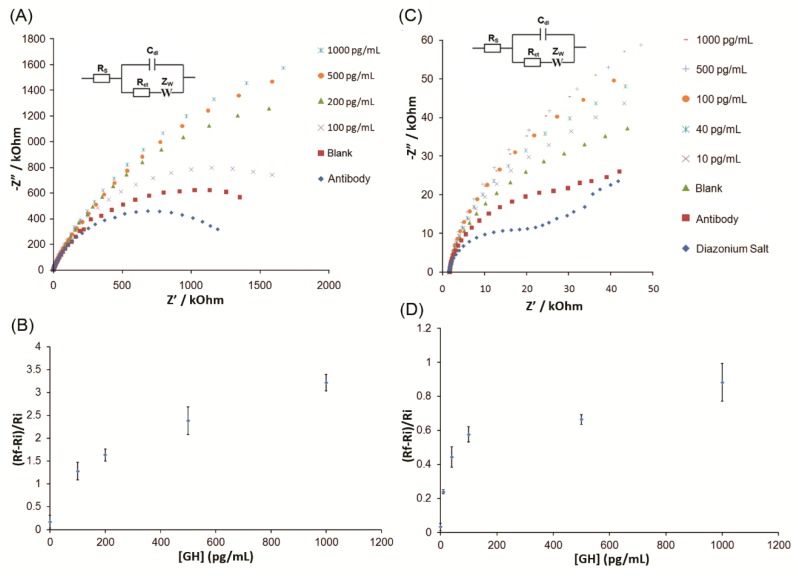
Impedimetric detection of growth hormone (GH) using antibody-modified carbon electrodes; (**A**) Nyquist plots for the detection of various concentrations of GH on glassy carbon electrodes (GCE); (**B**) Dependence of GH concentration on the R_ct_ values obtained from the Randles equivalent circuit at GCE. (**C**) Nyquist plots for the detection of various GH concentrations on screen-printed carbon electrodes (SPCE). (**D**) Dependence of GH concentration on the R_ct_ values obtained from the Randles equivalent circuit at the SPCE. Error bars indicate three consecutive measurements (n = 3) of each GH concentration using renewed GCE surfaces and using a new single-use SPCE.

**Figure 4:**
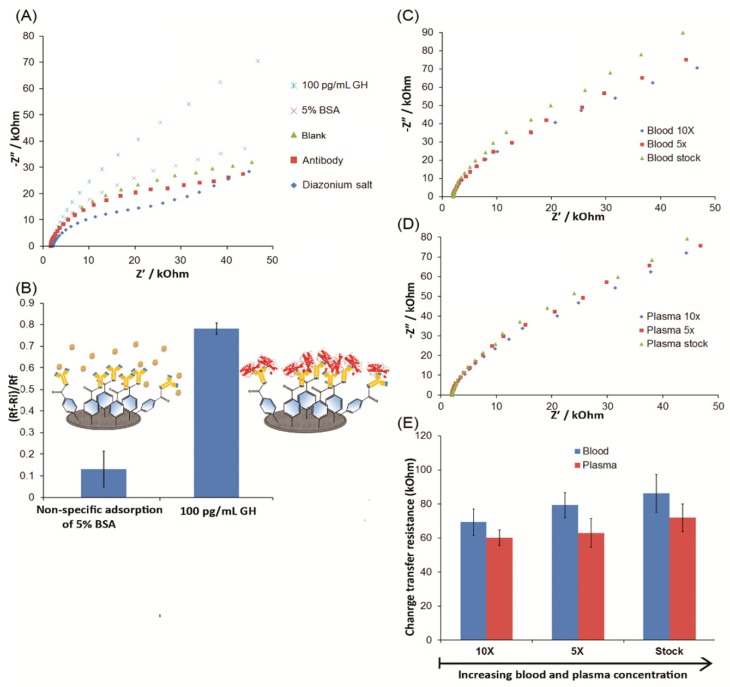
(**A**) Nyquist plots for the detection of growth hormone (GH) and bovine serum albumin (BSA) in diluted blood using screen-printed carbon electrodes (SPCE); (**B**) R_ct_ ratio comparison between attachment of BSA and GH in diluted blood (n = 3). (**C**) Nyquist plots for the detection of 100 pg/mL GH in various concentrations of blood. (**D**) Nyquist plots for the detection of 100 pg/mL GH in various concentrations plasma; (**E**) R_ct_ ratios for the detection of GH in various concentrations of blood and plasma (n = 3). Error bars indicate the standard deviation of three consecutive measurements using SPCEs (n = 3).

The anti-GH antibody-modified GCE enabled the detection of GH with a dynamic range between 100 pg/mL and 1000 pg/mL, as shown in Figure 3A. The initial (R_i_) and final (R_f_) R_ct_ values were measured before and after the GH binding to the antibody-modified electrodes. These R_ct_ ratios, between blank and a range of GH concentrations, are summarized in Figure 3B,D. The immunosensor was significantly improved using SPCEs with miniaturized and portable detection capabilities. As shown in Figure 3C, the R_ct_ values corresponding to each modification of the SPCEs continued to increase. The Nyquist plots increased with increasing concentrations of GH, and the R_ct_ ratios are displayed in Figure 3D. The linear range of GH detection using GCE was from 100 pg/mL to 1000 pg/mL, with a regression line formula y = 0.0021x + 1.1805, R^2^ = 0.982. Even though the dynamic range using SPCE was similar to that of the GCE surface, the linear range of GH detection was smaller, from 10 pg/mL to 100 pg/mL, with a regression line formula of y = 0.0035x + 0.2431, R^2^ = 0.9944. Using the formula for the limit of detection = 3 × σ_blank_/m, where σ_blank_ is the standard deviation of blank measurements and m is the slope of the calibration curve, we have determined the limit of detection as 5 pg/mL, which is highly comparable with literature values. Ozhikandathil et al. [38] reported a detection limit of 25 ng/mL using an evanescent-cascaded waveguide coupler design. Sadabadi et al. [39] reported the detection of GH using the LSPR response of gold nanoparticles grown in microfluidics with a detection limit of 3.7 ng/mL. In order to demonstrate the applicability of our GH detection method to biological samples containing high concentrations of potentially interfering proteins, a SPCE-based immunosensor was challenged with blood and plasma samples from male rats and the results are shown in Figure 4.

As shown in Figure 4A, the Nyquist plots displayed an increase with 4-MP film modification and the immobilization of antibodies. As shown in Figure 4B, BSA (5%, w/v) and GH (100 pg/mL) were spiked into 10-fold diluted blood samples. The Ri and Rf were measured before and after the binding of BSA or GH at the antibody-modified SPCEs. A p-value of 1.9 × 10^−5^ was calculated, by conventional criteria, the difference between R_ct_ ratios from the binding of BSA and the binding of GH was considered to be statistically highly significant. The R_ct_ ratio was significantly higher for GH-spiked blood samples, suggesting that the antibody-modified SPCEs were effective in facilitating the ultrasensitive detection of GH in the presence of complex protein mixtures present in blood. Hence, our immunosensor has promising potential for applications using clinical samples.

As shown in Figure 4C,D, GH (100 pg/mL) was detected in 10-fold and 5-fold diluted blood samples, as well as in the undiluted whole blood (blood stock) and plasma (plasma stock) samples. To investigate the reproducibility of our SPCEs, EIS responses of all 18 biosensors were recorded in these real samples that were spiked with 100 pg/mL GH, as shown in Figure 4E. The relative standard deviation was found to be less than 6% (n = 3 for each 10-fold and 5-fold diluted blood sample, as well as undiluted whole blood and plasma samples) with mean recoveries ranging from 94% ± 3% to 103% ± 2%. In Figure 4E, the average R_ct_ increased by 10 kOhm from 10-fold to 5-fold diluted blood samples, and 7 kOhm from the 5-fold diluted blood sample to the whole blood sample. This increase in R_ct_ was attributed to the possible non-specific adsorption of proteins in the whole blood matrix. The *p*-value was 4.6 × 10^−2^ between 10-fold and 5-fold diluted blood samples, and it was considered to be statistically significant by conventional criteria. However, the p-value was 2.4 × 10 between 5-fold diluted and whole blood samples, and it was not considered to be statistically significant by conventional criteria. In addition, the repeatability of the immunosensor was evaluated by performing three experiments with the similarly prepared three SPCEs for three consecutive days, and after each measurement, the SPCEs were disposed of. Prior to use, the immunosensors were soaked in 50 mM PBS (pH 7.4) and stored at 4 °C. SPCE-based immunosensors revealed a good repeatability performance with a relative standard deviation of less than 5% (n = 3 using 10-fold diluted plasma samples, data not shown).

Blood plasma samples with a simpler protein composition resulted in lower R_ct_ than whole blood samples in 10-fold diluted, 5-fold diluted and undiluted samples. The p-value was 4.6 × 10^2^ between 10-fold and 5-fold diluted plasma samples, and 8.6 × 10^2^ between 5-fold diluted and undiluted plasma samples. Both *p*-values were not considered to be statistically significant by conventional criteria and suggested increasing plasma concentrations did not result in a significant increase with non-specific adsorption and surface fouling.

For real sample studies, Vance et al. [40] have reported a comparative analysis of human growth hormone in serum using SPRi, nano-SPRi and ELISA assays. An ultrasensitive bimodal waveguide biosensor enabled the direct detection of human GH in undiluted urine samples in the 10 pg/mL range [41]. A nano-integrated suspended polymeric microfluidics platform provided a detection limit of 2 ng/mL for the detection of bovine growth hormones [42]. A summary of articles [43,44,45,46,47,48,49,50,51,52] reporting the detection of GH using various surface modifications and detection techniques with limits of detection is presented in Table 1. Rezaei et al. [45] modified gold electrodes with gold nanoparticles using 1,6-hexanethiol and managed to develop an ultrasensitive EIS-based immunosensor, which detected GH with a limit-of-detection of 0.64 pg/mL.

**Figure 5:**
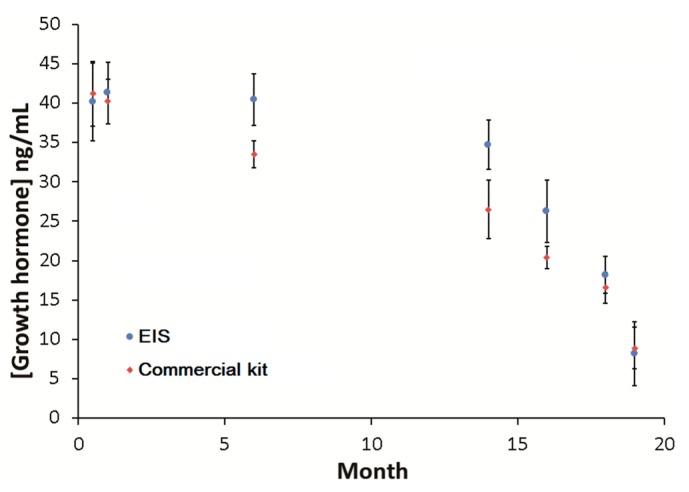
Dependence of growth hormone concentration on the age of rats. The results of EIS-based immunosensor using screen-printed carbon electrodes (SPCE) was compared with those obtained using the commercial detection kit as described in the Section 2.7. Error bars indicate the standard deviation of three consecutive measurements (n = 3) performed using the samples collected on the same day of each month.

Our immunosensor was applied to monitor GH in blood samples collected from male rats aged from 2 weeks to 19 months. EIS results of SPCEs were compared with those obtained using a commercial GH ELISA kit. As shown in Figure 5, GH levels decreased with advancing age rats using both detection systems. In general, the EIS data indicated that the rats had higher GH compared to the data obtained using the commercial kit. For the data that were obtained in months 1, 2, 18 and 19 using EIS-based immunosensors and commercial kit, the *p*-values ranged between 2.7 × 10^2^ and 4.3 × 10^2^. These *p*-values were not considered to be statistically significant by conventional criteria and suggested that the data collected in those months were similar. In months 6, 14 and 16, the fluctuation of GH created data with *p*-values ranging between 3.5 × 10^−4^ and 1.8 × 10^−3^, indicating that the data were significantly different from each other. This was attributed to the possible fluctuations of GH in adult rats during these months. The lower GH concentration determined by the commercial kit may be attributed to the complicated sample preparation procedures, as well as the short lifetime of the light-sensitive TMB (3,3′,5,5′-tetramethylbenzidine) dye, which was observed to decrease in absorbance rapidly over time.

## 4. Conclusions

An immunosensor was developed using aryl diazonium salt modification for the covalent immobilization of antibodies on screen-printed carbon electrodes (SPCE) and glassy carbon electrode (GCE) surfaces. SPCE-based immunosensors provided the development of low-cost and single-use detection systems, avoiding possible cross-contamination issues that can be observed using GCE surfaces. The impedimetric detection of GH exhibited a high analytical performance, demonstrating good sensitivity with good reproducibility and repeatability. The results demonstrated that the immunosensor could detect 5 pg/mL GH with a linear range between 10 pg/mL and 100 pg/mL GH. The detection of GH in the presence of increasing concentrations of complex protein mixtures present in blood demonstrated the applicability of the technique to real samples.

## Figures and Tables

**Table 1:** A summary of published literature on the detection of GH using biosensors with optical and electrochemical detection techniques.

Biosensor Surface	Detection Technique	Limit of Detection	Reference
Silica-on-silicon (SOS) with a cascaded waveguide coupler	Evanescent wave-based fluoroimmunoassay	25 ng/mL	[38]
Gold nanoparticles synthesized in a poly(dimethylsiloxane) (PDMS) microfluidic chip	LSPR-based immunoassay	3.7 ng/mL	[39]
Anti-hGH coated with near-infrared quantum dots	SPRi (SPR imaging)- & Nano-SPRi-based immunoassay	0.03 ng/mL–100 ng/mL	[40]
Anti-hGH-modified interferometer	Bimodal waveguide interferometry-based immunoassay	10 pg/mL	[41]
Nano-integrated suspended polymeric microfluidics (SPMF) platform	Microcantilever-based immunoassay	2 ng/mL	[42]
Anti-hGH modified gold surfaces	SPR-based immunoassay	6 ng/mL	[43]
Anti-hGH modified gold surfaces	SPR-based immunoassay	1-6 ng/mL	[44]
Gold nanoparticles immobilized on gold electrodes using 1,6-hexanedithiol	EIS-based immunoassay	0.64 pg/mL	[45]
Sandwich-based immunoassay using horseradish peroxidase (HRP)-labeled secondary antibody	SPR, pulsed amperometry (PA), electrochemically-assisted chemiluminescence (ECL), CV	0.051 nM by SPR 0.027 nM by PA 0.061 nM by ECL 0.056 nM by CV	[46]
Tosyl-activated magnetic microparticles on screen-printed gold electrodes	Square-wave voltammetry (SWV) of 4-aminophenyl phosphate as the substrate of alkaline phosphatase	0.005 ng/mL	[47]
Protein A-gold binding domain fusion protein	SPR-based immunoassay	90 ng/mL	[48]
Anti-hGH immobilized on gold surfaces	PA & CV	75 nM by PA 108 nM by CV	[49]
Oriented anti-hGH-modified gold surfaces using biotin-streptavidin	SPR-based immunoassay	0.9 ng/mL for 22K and 20K hGH isoforms	[50]
Plasmonic gold decorated multi-walled carbon nanotube nanocomposite	LSPR-based immunoassay	1 ng/mL	[51]
Carbon fiber microelectrode	Differential pulse voltammetry for in vivo and ex vivo measurements using rats	2 µg/µL	[52]
Anti-GH modified SPCE	EIS-based immunoassay	5 pg/mL	This work
